# Autosomal Recessive Hypercholesterolemia Caused by a Novel *LDLRAP1* Variant and Membranous Nephropathy in a Chinese Girl: A Case Report

**DOI:** 10.3389/fcvm.2022.811317

**Published:** 2022-02-04

**Authors:** Siqin Feng, Xinyue Zhao, Yifei Wang, Yiyang Wang, Gang Chen, Shuyang Zhang

**Affiliations:** ^1^Department of Cardiology, Peking Union Medical College Hospital and Chinese Academy of Medical Sciences and Peking Union Medical College, Beijing, China; ^2^School of Medicine, Tsinghua University, Beijing, China; ^3^Department of Cardiology, Beijing Tsinghua Changgung Hospital, Tsinghua University, Beijing, China; ^4^Department of Nephropathy, Peking Union Medical College Hospital, Beijing, China

**Keywords:** nephrotic syndrome, membranous nephropathy, homozygous familial hypercholesterolemia, *LDLRAP1*, hyperlipidemia

## Abstract

**Background:**

Autosomal recessive familial hypercholesterolemia (ARH) is a very rare lipid metabolic monogenic disorder caused by homozygosity or compound heterozygosity for mutations in the low-density lipoprotein receptor adapter protein 1 (*LDLRAP1*) gene. It is a life-threatening disease characterized by markedly elevated low-density lipoprotein cholesterol (LDL-C), xanthomas, and premature coronary artery disease. Membranous nephropathy (MN) is less commonly observed in children. Here, the co-existence of ARH and MN was diagnosed in a Chinese girl.

**Case Presentation:**

We present the case of a 13-year-old girl who was admitted with the typical symptom of nephrotic syndrome with an abnormally high serum LDL-C level. Gene sequencing revealed a novel homozygous *LDLRAP1* variant (NM_015627: c.383 T>G, p.V128G), and the patient was diagnosed with ARH. A renal biopsy suggested that the nephrotic syndrome in the girl was induced by MN, but no evidence of secondary MN was found. A thorough examination was performed to explore the association between MN and ARH. Medical management with angiotensin receptor blockers and aggressive lipid-lowering treatment led to remission of proteinuria and clinical condition stabilization during 2-year follow-up.

**Conclusions:**

This is the first case of co-existence of MN and ARH in a teenager carrying a novel pathogenic mutation of the *LDLRAP1* gene (NM_015627: c.383 T>G, p.V128G).

## Introduction

Autosomal recessive familial hypercholesterolemia (ARH) (OMIN #603813) is a very rare monogenic lipid metabolic disorder affecting <1 in every 1,000,000 people, and is characterized by significantly elevated low-density lipoprotein cholesterol (LDL-C) levels and premature life-threatening atherosclerotic cardiovascular disease (ASCVD) (1, 2). ARH is induced by mutations in the LDL receptor adaptor protein 1 *(LDLRAP1)* gene, which is located in the short arm of chromosome 1 at position 36.11 (1p36.11) and provides instructions for synthesizing LDLRAP1, an essential protein for the endocytosis of LDL. Without the LDLRAP1 protein, excessive plasma LDL-C deposits in the cornea, skin, tendons, and arteries form cutaneous or tendon xanthomas in the corneal arcus, leading to early-onset and rapidly progressing atherosclerosis.

Membranous nephropathy (MN) is a pathologically defined disorder of the kidney glomerulus featuring glomerular basement membrane (GBM) thickening with little or no cellular proliferation or infiltration (3). Nephrotic syndrome (NS) is the bread and butter of nephrology referrals, and MN is among the most common causes of NS in non-diabetic adults but less commonly seen in children, in whom it is often associated with non-steroidal anti-inflammatory drug use, hepatitis B or hepatitis C, or, less commonly, autoimmune thyroid disease, and malignancies. NS is characterized by proteinuria and hypoalbuminemia with or without edema and hyperlipidemia, and the association between hyperlipidemia and NS remains controversial.

The “lipid nephrotoxicity” hypothesis states that hyperlipidemia may lead to proteinuria, glomerulosclerosis, and other injurious effects on the kidneys (4, 5). The association between extremely abnormal dyslipidemia and nephropathy is not completely understood. In clinical practice, the comorbid presence of classical NS and extremely abnormal dyslipidemia makes diagnosis difficult.

Here we report the case of a newly discovered *LDLRAP1* variant of ARH accompanied by MN-induced NS and followed the patient's clinical and laboratory findings and response to medical management over 2 years.

## Case Description

**Figure 1:**
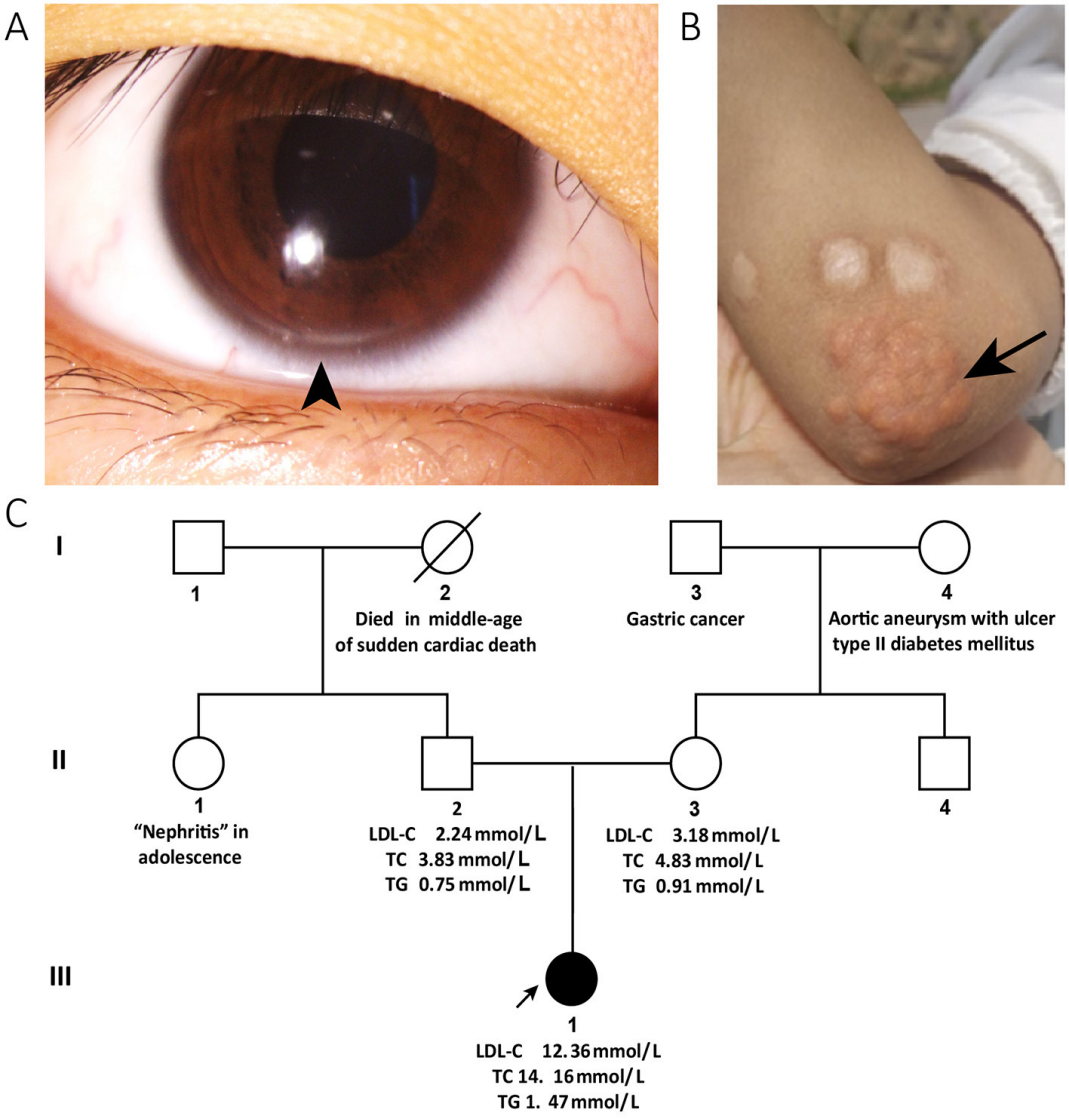
Clinical characteristics and pedigree of the patient. The corneal arcus (arrowhead) is visible in the inferior margin of the iris **(A)** and cutaneous xanthomas (arrow) in the elbow **(B)**. **(C)** Shows the pedigree of the patient: the circles indicate female family members, squares indicate male family members, slash denotes deceased members, solid symbols indicate affected family members, and open symbols denote unaffected family members. The proband is a family member, III-1. The data of the patient's blood lipids were derived from tests taken during her first visit to our clinic, while those of her parents were derived from the results of a physical examination within a year. The patient's paternal aunt reportedly developed “nephritis” in adolescence. Her paternal grandmother died of sudden cardiac death in middle age. Her maternal grandmother developed an aortic aneurysm with ulcers, and type II diabetes mellitus. Her maternal grandfather had a history of gastric cancer. LDL-C, low-density lipoprotein cholesterol; TC, total cholesterol.

A 13-year-old girl with a 4-month history of periorbital edema was referred to our clinic in December 2019. The periorbital edema of the patient appeared bilaterally every morning with no obvious inducements and disappeared half an hour later without eye redness, heat, or pain and was accompanied by intermittent foamy urine. No other specific discomforts, such as gross hematuria, fever, backache, rash, lower limb edema, or suspicious drug use were reported. The blood pressure of the patient was 104/67 mmHg, heart rate was 92 beats/min, and body mass index was 15.24. She appeared thin and sallow, with bilateral arcus corneas (Figure 1A) and multiple xanthomas located on the extensor sides of the right thumb, bilateral elbows, and knee joints (Figure 1B) that first appeared at 3 years of age. Physical findings of other relevant systems were normal.

The patient's paternal aunt reportedly developed “nephritis” in adolescence. Her paternal grandmother died of “sudden cardiac death” in middle age. Her maternal grandmother developed an aortic aneurysm with ulcers, and type II diabetes mellitus. Her maternal grandfather had a history of gastric cancer. There was no familial history of xanthomas, corneal arcus, or hyperlipidemia. The pedigree of the proband's family is shown in Figure 1C.

**Figure 2:**
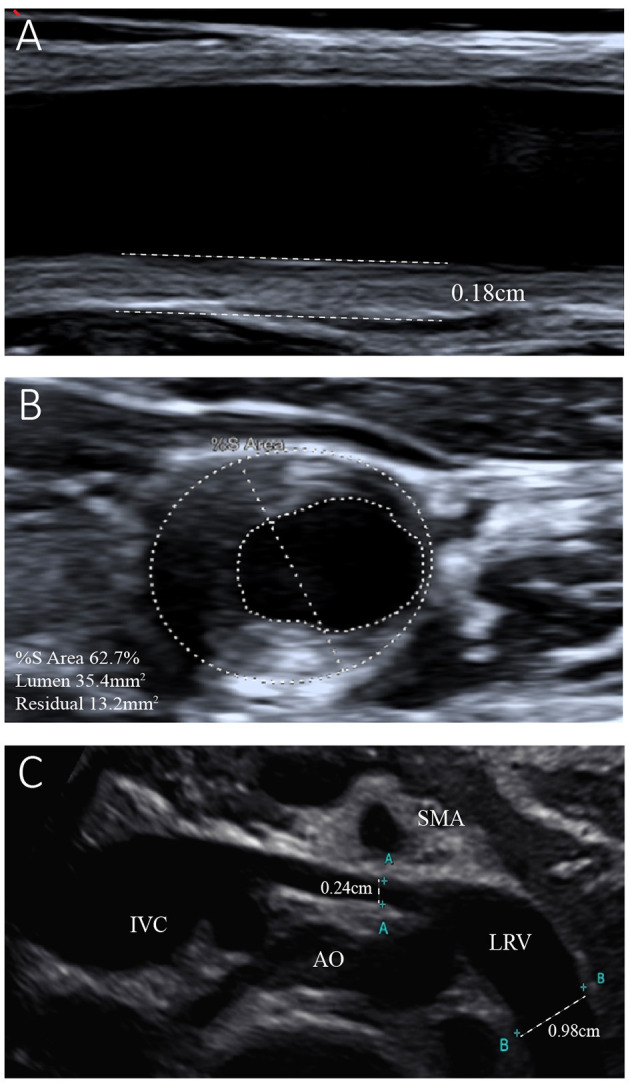
Ultrasonogram of the left common carotid artery and left renal vein. **(A)** Reveals left carotid atherosclerosis with plaque formation and intima-media thickness of the left common carotid artery of 0.18 cm. **(B)** Shows 62.7% stenosis of the left common carotid artery. **(C)** Shows entrapment of the left renal vein (LRV) between the aorta (AO) and superior mesenteric artery (SMA). The internal diameter of the LRV was 0.24 cm, while that of the distal segment was 0.98 cm.

**Figure 3:**
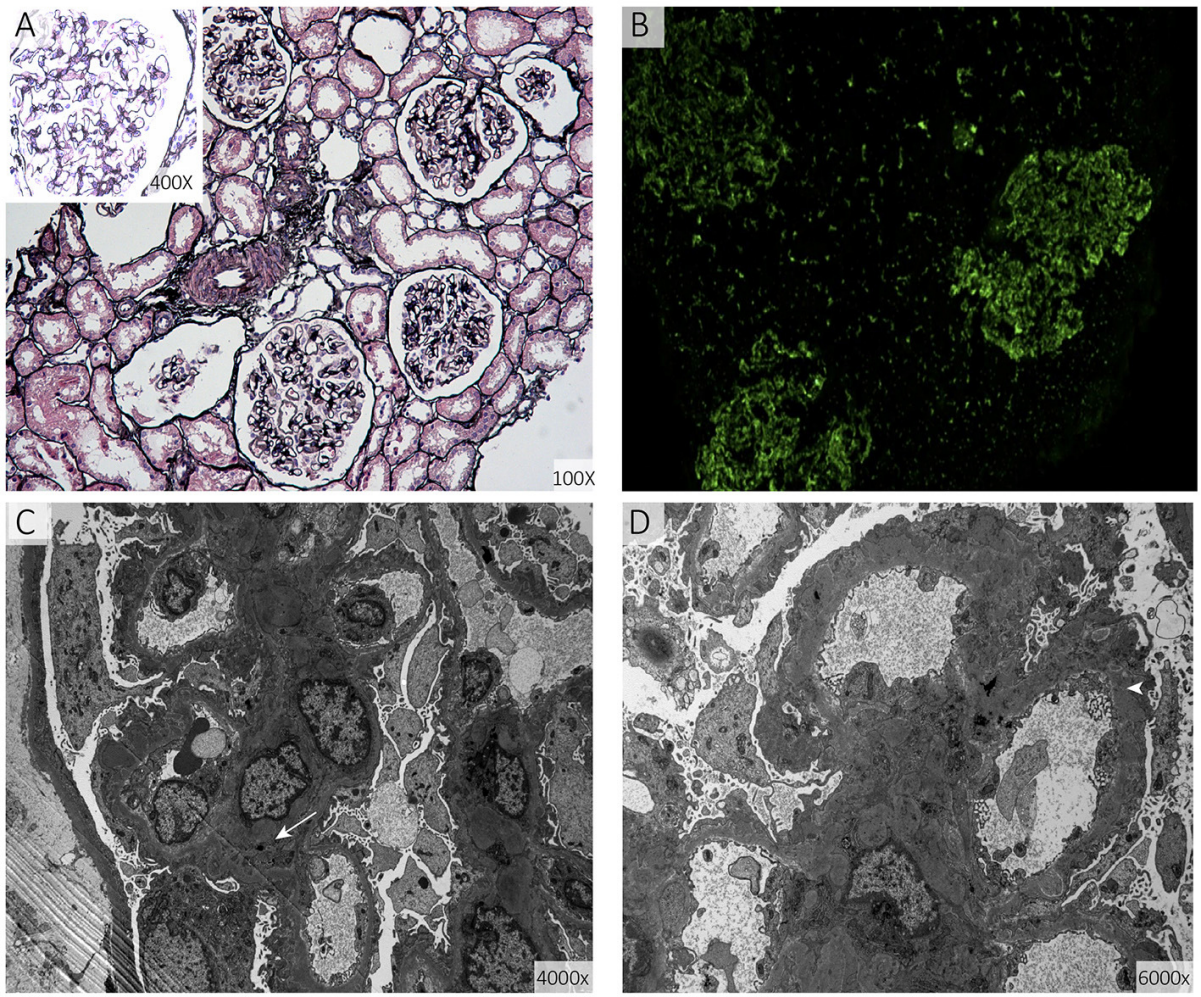
Renal biopsy specimens. **(A)** Shows diffuse thickening of the basement membrane of the glomerulus together with spike formation (periodic acid-silver methenamine). **(B)** Shows diffuse granular deposits in the vascular loops (direct immunofluorescence staining for immunoglobulin G). **(C,D)** show diffuse irregular thickening of the basement membrane and electron-dense deposits in the basement membrane (arrow) and sub-epithelial area (arrowhead) (electron micrograph of the glomerulus).

The results of the girl's laboratory tests were as follows (partly shown in Table 1): total cholesterol 14.65 mmol/L; LDL-C 12.92 mmol/L; high-density lipoprotein cholesterol (HDL-C), 0.93 mmol/L; triglyceride 1.68 mmol/L; apolipoprotein B 3.85 g/L; apolipoprotein A1 1.14 g/L; lipoprotein(a) 602.2 mg/L; creatinine 19 μmol/L; estimated glomerular filtration rate 193.83 ml/min/1.73 m^2^; urea nitrogen 2.1 mmol/L; and albumin, 29 g/L. A dipstick urinalysis showed hematuria (2+) and proteinuria (2+); urine sediment showed a few red cells, 60% of which were normal; and urinary protein was 7.25 g/24 h. No malignancies or thyroid disease was discovered, and other routine blood examinations, such as anti-PLA2R, antinuclear, anti-double-stranded DNA, anti-Smith, anti-RNP, and anti-neutrophil cytoplasmic antibodies, were all negative. Complements C3 and C4, immunoglobulin, rheumatoid factor, and anti-streptolysin levels were within normal range. She was seronegative for human immunodeficiency virus and hepatitis B and C viruses. Electrocardiography and echocardiography indicated normal cardiac structure and function. Ultrasonography of the arteries showed atherosclerotic plaques in the bilateral carotid arteries (Figures 2A,B), subclavian artery, innominate artery bifurcation, and abdominal aorta. Ultrasound of the urinary system showed no abnormalities, while that of the renal veins showed entrapment of the left renal vein between the aorta and superior mesenteric artery, consistent with nutcracker syndrome (Figure 2C). To further elucidate the cause of the high urinary protein level, a renal biopsy was performed. Immunofluorescence microscopy revealed diffuse granular deposition of IgG++, C3++, IgG1 +-++, IgG2 ++, IgG3 +, and PLA2R + + ++ in vascular areas. GBM was diffusely thickened, and spike formation was observed on the epithelial side. Granules and balloon-cell degeneration were visible in the renal tubular epithelial cells. No obvious abnormalities were observed in the interstitium or small blood vessels. In addition, no lipid deposition was observed, and no significant staining of Oil Red O was noted (Figure 3). The pathology of renal biopsy demonstrated MN (stage II), but atypical membranous nephropathy might not be excluded, with electron-dense deposition in the subepithelial area, basement membrane, and mesangial zone.

**Table 1:** Dynamics of laboratory tests, key highlights, and treatment.

**Patient's age**	**TC**	**LDL-C**	**TG**	**HDL-C**	**Alb**	**Cr**	**24 hUP**	**Key highlights**
**(date: dd/mm/yyyy)**	**(2.80–5.70 mmol/L)**	**(2.7–3.1 mmol/L)**	**(0.29–1.83 mmol/L)**	**(1.16–1.55 mmol/L)**	**(40–55 g/L)**	**(53–115 μmol/L)**	**(<0.15 g/24 h)**	**and treatment**
13y (10-12-2019)	14.16	12.36	1.47	0.76	31	39	-	Genetic screening and renal biopsy were conducted in our hospital. Treatment with atorvastatin (30 mg/day), ezetimibe (10 mg/day) and benazepril (10 mg/day) were started. Administration of PCSK9 inhibitor evolocumab (420 mg/month) was started 1 month after discharge.
13y (27-12-2019)	13.03	9.84	1.82	1.09	34	36	2.08	
14y (06-06-2020)	8.55	6.14	1.11	1.85	66	25	-	Rosuvastatin (10 mg/day) was used to replace atorvastatin.
14y (07-09-2020)	13.05	10.2	1.03	2.15	69.9	26	0.01	Patient discontinued rosuvastatin (10 mg/day) and benazepril was stopped.
14y (04-12-2020)	7.36	5.36	1.12	1.57	70.8	33	0.01	15 mg atorvastatin was administered daily.
15y (06-03-2021)	6.36	4.81	0.94	1.22	42.9	-	0.08	Medication is composed of atorvastatin (15 mg/day), ezetimibe (10 mg/day), PCSK9 inhibitor evolocumab (420 mg/month).
15y (18-09-2021)	6.29	4.63	0.60	1.25	44.5	41.90	-	

*Exact dates of the clinic visits and drug administration were recorded. 24 hUP, 24-h urinary protein; Alb, albumin; Cr, creatinine. To convert total cholesterol (TC), low-density lipoprotein cholesterol (LDL-C) and high-density lipoprotein cholesterol (HDL-C) from millimoles per liter to milligrams per deciliter, the value was divided by 38.67. To convert triglycerides (TGs) from millimoles per liter to milligrams per deciliter, the value was divided by 88*.

A whole-exome sequencing analysis of the genomic DNA of the patient and her parents was performed. The proband carried a novel homozygous *LDLRAP1* gene variant, NM_015627: c.383 T>G, p.V128G, which was not reported in the database of the Leiden Open-Source Variation Database, Human Gene Mutation Database, or previous literature. Her parents, who were verified as *LDLRAP1* heterozygotes, denied being consanguineous but were from the same village.

Upon completion of all the examinations mentioned above at Peking Union Medical College Hospital, the diagnoses of ARH, MN-induced NS, and nutcracker syndrome were confirmed. The girl then received detailed nutritional guidance as follows: her overall energy requirement was about 1,600 kcal/day, and protein was 60–80 g/day. Reduced intake of saturated fatty acids and recommended intake of exogenous cholesterol were 20%, as were reduced sugar intake and reduced high-trans fatty acid intake. Treatment with the angiotensin receptor blocker benazepril (10 mg/day), atorvastatin (30 mg/day), and ezetimibe (10 mg/day) was initiated, and the PCSK9 inhibitor evolocumab (420 mg/month) was initiated 1 month after discharge. The girl and her parents rejected the offer of lipoprotein apheresis and the possibility of liver transplantation.

The dynamics of the laboratory tests, key highlights, and response to treatment for the next 2 years are shown in Table 1. Within 10 months of aggressive LLT along with continuation of angiotensin receptor blocker, she responded very well with improvement in 24-h urinary protein from 2.08 g to only 0.01 g (99.9% reduction) and stopped benazepril administration. During the follow-up, she sustained aggressive LLT, including atorvastatin (15 mg/day), ezetimibe (10 mg/day), and PCSK9 inhibitor evolocumab (420 mg/month). Her blood total cholesterol (TC), LDL-C, and lipoprotein(a) decreased to 6.29 mmol/L (55.6%), 4.63 mmol/L (62.5% reduction), and 118 mg/L (80.4% reduction), respectively, which even led to regression of the large xanthomas.

## Discussion

Here, we reported the case of a patient with a homozygous novel variant of the *LDLRAP1* gene (NM_015627: c.383 T>G, p.V128G) causing ARH, the first case of the co-existence of ARH and MN. To date, 20 pathogenic variants have been identified in the coding sequence of the *LDLRAP1* gene, leading to the impairment of cholesterol metabolism and development of clinical signs of hypercholesterolemia. The extreme rarity of the variant and its homozygous state encouraged us to classify it as probably pathogenic. The clinical picture of ARH mimics that of homozygous familial hypercholesterolemia (HoFH), and our patient presented with a significant phenotypic FH manifestation at an extremely young age. In such cases, aggressive and prompt lipid-lowering medications are invariably required along with lifestyle improvement, lipoprotein apheresis, liver transplantation, and additional new therapeutic options such as gene therapy. However, despite the adoption of a variety of combination drug therapies, the LDL-C levels of most patients with ARH and HoFH do not reach the recommended goals, thereby predisposing them to cardiovascular diseases (6).

Membranous nephropathy (MN), a leading cause of primary NS in adults, is rare in children. In addition, 70–80% of patients with primary MN have autoantibodies against the M-type phospholipase A2 receptor (PLA2R), which is associated with decreased renal function and poorer prognosis (7). Patients with MN and both negative glomerular PLA2R and serum anti-PLA2R antibodies usually have lower 24-h urinary protein levels and higher remission rate than those with MN and both positive antibodies, which may partially explain the remission of our patient.

The causality between nephropathy and hypercholesterolemia warrants further investigation. Nephropathy may be induced by severe hyperlipidemia, because previous studies have demonstrated a link between hyperlipidemia and glomerular injury (8, 9). In very rare cases, some genetic defects related to lipid metabolism, such as Tangier disease (10) and lecithin-cholesterol acyltransferase deficiency (11), can also lead to renal lipotoxicity. A case of HoFH and focal segmental glomerulosclerosis was also reported (12). However, the results of renal vessel ultrasonography and histopathology of renal biopsy did not reveal renal arteriosclerosis, hyaline degeneration of the small vessels, or glomerular lipid deposition. A renal biopsy revealed classical MN with limited damage induced by hyperlipidemia. From a clinical perspective, this may indicate that ARH and MN-related NS, two rare diseases in children, occurred independently and coincidently in the 13-year-old girl. Nutcracker syndrome was also identified, which also partially explained the proteinuria and hematuria that occurred in our thin patient. At the same time, LDLRAP1 is far more an adaptor protein that interacts with the cytoplasmic tail of LDLR, phospholipids, and components of the clathrin endocytic machinery, and controls LDL-C levels. It was reported to regulate the endocytosis of renal outer medullary potassium (ROMK) channels in mouse kidneys, which may contribute to potassium retention and hyperkalemia (13). Studies using large-scale genome-wide association study (GWAS) suggested that genetic variation of *NFKB1, IRF4, PLA2R1*, and *HLA* were interacted with the primary MN (14). Evidence for the independent nature of MN in HoFH or ARH background is not rock-solid and mechanistic studies are lacking. The possibility that the mutation in LDLRAP1 is the causative mutation of both MN and FH remains to exist and is worthy of further exploration.

Considering the normal renal function of the patient, we offered only benazepril and aggressive lipid-lowering treatment. Nutritional guidance should be offered to improve the nutritional status of the patient. The remission of NS might have partially been a result of well-controlled serum cholesterol and improved body nutrition. We initially assumed that NS was induced by extremely abnormal hyperlipidemia. This is a very rare phenomenon described in only few reports. However, a renal biopsy revealed that the actual cause of the symptoms of the patient was far rarer than expected. MN, a rare pathological change in children, and ARH, a rare disease induced by *LDLRAP1* mutation, contributed to the symptoms of this 13-year-old girl in a “rare plus rare” manner.

### Patient Perspective

Autosomal recessive familial hypercholesterolemia (ARH) is a very rare lipid metabolic monogenic disorder characterized by markedly elevated levels of LDL-C, xanthomas, and premature coronary artery disease. From this perspective, its early diagnosis and management could reduce significant cardiovascular events that may lead to life-threatening events.

The patient herself had felt self-abased because of the xanthoma and was afraid to wear short sleeves in public. Besides, the pathologic changes that had occurred in the girl's kidney restricted her from going to school or performing vigorous physical activities. After the early diagnosis and long-term lipid-lowering treatment, the xanthoma gradually subsided, and she was able to return to school after the remission of foamy urine and participate in various social activities with more confidence.

## Data Availability Statement

The original contributions presented in the study are included in the article/supplementary material, further inquiries can be directed to the corresponding authors.

## Ethics Statement

The studies involving human participants were reviewed and approved by the Ethics Committee of Peking Union Medical College Hospital (JS1195) and performed in accordance with the Declaration of Helsinki. Written informed consent to participate in this study was provided by the participants' legal guardian/next of kin. Written informed consent was obtained from the minor(s)' legal guardian/next of kin for the publication of any potentially identifiable images or data included in this article. Signed informed consent was obtained from the parents of the patient for her participation and the sample use in this study.

## Author Contributions

SF conceived the study, contributed to the data collection, approved the final version, and is accountable for its accuracy and integrity. XZ and YiyW wrote the first draft and revised the subsequent drafts. SF, XZ, and YifW contributed to the patient's diagnosis and treatment. GC was responsible for the results and interpretation of the renal pathology. GC and SZ contributed to the approval of the final version and take accountability for the accuracy and integrity of the findings. All authors have read and approved the manuscript for publication.

## Funding

This study was supported by the Beijing Natural Science Foundation (Grant No: L202046) and the National Natural Science Foundation (Grant No: 8217022134) to cover the cost of the whole-exome sequencing.

## Conflict of Interest

The authors declare that the research was conducted in the absence of any commercial or financial relationships that could be construed as a potential conflict of interest.

## Publisher's Note

All claims expressed in this article are solely those of the authors and do not necessarily represent those of their affiliated organizations, or those of the publisher, the editors and the reviewers. Any product that may be evaluated in this article, or claim that may be made by its manufacturer, is not guaranteed or endorsed by the publisher.
